# Chirality-driven all-optical image differentiation

**DOI:** 10.1515/nanoph-2025-0479

**Published:** 2025-12-09

**Authors:** Stefanos Fr. Koufidis, Zeki Hayran, Francesco Monticone, John B. Pendry, Martin W. McCall

**Affiliations:** Blackett Laboratory, Department of Physics, Imperial College of Science, Technology and Medicine, Prince Consort Road, London SW7 2AZ, UK; School of Electrical and Computer Engineering, Cornell University, Ithaca, NY 14853, USA

**Keywords:** analog computing, chirality, image differentiation, Laplacian, metamaterials, spectral engineering

## Abstract

Optical analog computing enables powerful functionalities, including spatial differentiation, image processing, and ultrafast linear operations. Yet, most existing approaches rely on resonant or periodic structures, whose performance is strongly wavelength-dependent, imposing bandwidth limitations and demanding stringent fabrication tolerances. Here, to address some of these challenges, we introduce a highly tunable platform for optical processing, composed of two cascaded uniform slabs exhibiting both circular and linear birefringence, whose response exhibits features relevant to optical processing without relying on resonances. Specifically, using a coupled-wave theory framework we show that sharp reflection minima, referred to as spectral holes, emerge from destructive interference between counter-propagating circularly polarized waves in uniform birefringent slabs, and can be engineered solely through parameter tuning without requiring any spatial periodicity. When operated in the negative-refraction regime enabled by giant chirality, the interference response acquires a highly parabolic form around the reflection minimum, giving rise to a polarization-selective Laplacian-like operator that performs accurate spatial differentiation over a broad spatial-frequency range. This functionality is demonstrated through an edge-detection proof of concept. The required material parameters align closely with recent experimental demonstrations of giant, tunable chirality via meta-optics, presenting a promising pathway towards compact and reconfigurable platforms for all-optical pattern recognition and image restoration.

## 1 Introduction

Optical analog computing harnesses the wave nature of light to execute mathematical operations via passive, parallel physical processes, offering significant reductions in energy consumption and latency compared to conventional electronic computation, despite similar underlying signal speeds [1]. Early demonstrations showed that engineered meta-media could realize spatial operations such as edge detection and convolution by encoding mathematical kernels into the local amplitude and phase responses of planar nanostructures [2], [3], with more recent implementations spanning from dispersion-engineered metasurfaces [4] to nonlinear thin films [5]. These passive, planar platforms carry out complex computations directly on incident wavefronts, highlighting their potential for real-time image processing, neuromorphic photonics, and other applications that exploit inherent spatial parallelism [6].

Programmable and reconfigurable optical platforms have also recently been developed that can implement arbitrary linear – and even nonlinear – transformations, such as matrix inversion and root finding, thereby broadening the computational repertoire of wave-based processors [7], [8]. Temporal metamaterials, in turn, provide a fundamentally distinct platform for analog computing, drawing on nonlocal effects [9] or spin-controlled dynamics [10] to achieve first-order waveform differentiation.

Complementary to these developments, analog optical operations have also been demonstrated using a range of other photonic mechanisms, often relying on distributed-feedback effects to manipulate the phase response of propagating light. Techniques based on phase-shifted gratings have enabled first- and higher-order differentiation and integration by embedding discrete phase discontinuities into otherwise homogeneous media [11], [12], [13]. Such methods have been successfully adapted to THz-compatible devices using silicon-on-insulator directional couplers [14] and reconfigurable interferometric signal processors capable of performing versatile linear operations on-chip [15]. Related approaches involving phase-shifted Bragg gratings, plasmonic waveguides, or spatially dispersive metasurfaces composed of anisotropic unit cells [16] have enabled beam-profile differentiation by selectively reflecting angular field components, effectively implementing spatial operations in the Fourier domain [17], [18], [19], [20]. Nonetheless, the reliance of these systems on resonant or periodic structures restricts their operation to narrow spectral bands and precise incidence angles, limiting their potential for general-purpose broadband computing. These constraints also impose demanding fabrication tolerances, hindering scalability and reconfigurability.

In this communication we propose a parameter-tunable, non-periodic mechanism for achieving broadband Laplacian-type differentiation in modulus, by cascading two uniform slabs of chiral-birefringent media. Each slab combines circular birefringence, characterized by a magnetoelectric chirality parameter *α*, with linear dielectric anisotropy. By introducing a slight mismatch between the average refractive indices of the slabs, we derive explicit conditions under which destructive interference generates a sharp reflection dip – a “spectral hole” – in the chirality domain. Coupled-wave analysis reveals that at this spectral hole the magnitude of the reflection coefficient exhibits a parabolic dependence on the transverse wavenumbers, *k*_*x*_ and *k*_*y*_, thereby yielding a transfer function proportional to $k_{x}^{2}+k_{y}^{2}$ , the hallmark of Laplacian-like differentiation, over a broad spectral range.

The required parameters align closely with recent advances in metamaterials [21] and metasurfaces [22] exhibiting giant and controllable chirality [23]. Consequently, our approach yields compact, reconfigurable photonic differentiators that are not subject to the narrowband spectral response imposed by the resonant wavelength dependence of periodic structures governed by the Bragg condition. As with all related methods discussed above, our approach is naturally suited for operation with spatially coherent light [24].

The manuscript is organized as follows: After revisiting the eigenmodes of circularly and linearly birefringent media, detailed in Appendix A, Section 2 delineates the conditions necessary for generating high-quality spectral holes in the chirality domain, as opposed to the traditional wavelength domain. Subsequently, Section 3 evaluates the functionality and performance of the proposed polarization-selective spatial differentiator, demonstrating the excellent parabolic fit of the transfer function and illustrating its utility in edge detection applications. Furthermore, by accounting for realistic material dispersion, we directly compare the proposed scheme with established Bragg-based devices, demonstrating its superior performance in terms of angular range. Section 4 then discusses practical implementations in current meta-media that satisfy the broadband operational criteria, followed by Section 5 which summarizes our key findings.

## 2 Chiral spectral holes

**Figure 1:**
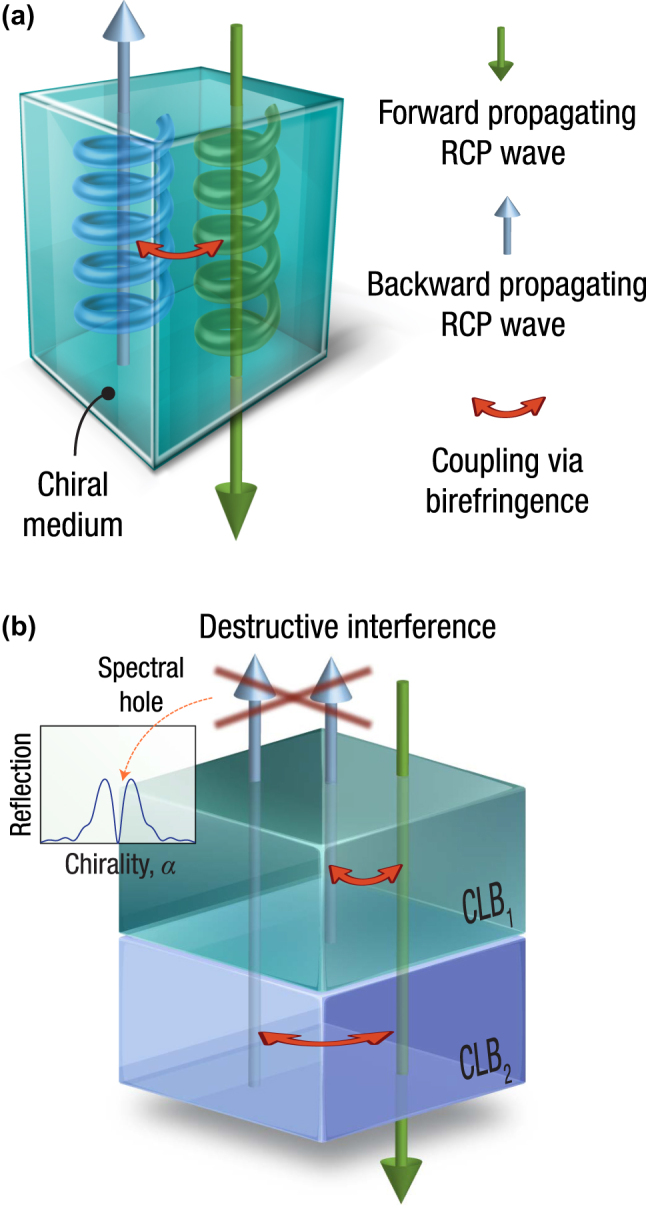
Chirality-induced reflection effects in birefringent media. (a) Interaction between forward- and backward-propagating circularly polarized waves in a chiral medium, mediated by linear birefringence, gives rise to the notion of a “grating-less grating” reported in Ref. [25]. (b) The structure under discussion consists of two *uniform* slabs of equal thickness, labeled CLB_1_ and CLB_2_, each exhibiting both circular birefringence and transverse dielectric anisotropy. Under corresponding preconditions, a slight mismatch in their average refractive indices leads to destructive interference between counter-propagating modes. This interference results in a reflection minimum that appears in the chirality domain, governed entirely by material parameter tuning rather than spatial periodicity. Since this response does not originate from a conventional resonance, the effect is not constrained by a narrow resonance bandwidth, but only by material dispersion.

Traditionally, the concept of all-optical spatial differentiation has been primarily associated with the formation of spectral holes in the optical spectrum of cascaded, spatially modulated Bragg gratings [17], [18]. At the core of Bragg gratings lies the Bragg condition, whereby incident lightwaves whose wavelength matches the pitch of the grating are strongly backscattered, as successive reflections coherently superimpose. However, Ref. [25] introduced a distinct yet physically related mechanism that arises in *homogeneous* media exhibiting both circular and linear birefringence (CLB), thereby lifting the requirement for wavelength matching – see Figure 1(a). Owing to its dependence solely on material parameters, the recently identified Bragg-like effect is intrinsically broadband, limited only by material dispersion.

A schematic depiction of a configuration capable of supporting chiral spectral holes is shown in Figure 1(b): it comprises two cascaded, homogeneous slabs – CLB_1_ and CLB_2_ – each exhibiting both circular and linear birefringence. A circularly polarized wave of a given handedness, say right, is normally incident onto the system and is partially coupled into a backward-propagating wave through birefringence-induced mixing. As demonstrated in the analysis below, a slight mismatch between the average refractive indices of the two slabs ensures destructive interference between the counter-propagating waves. Such an interference produces a pronounced reflection minimum – i.e., a spectral hole – that originates from the intrinsic material parameters and the slab length, rather than from any wavelength-scale periodic modulation.

To begin with, let us consider a medium characterized by relative permittivity *ϵ*_*r*_, permeability *μ*_*r*_, and chirality parameter *α*. The latter quantifies magneto-electric coupling, inducing circular birefringence by shifting the refractive indices of right- and left-circular polarizations (RCP and LCP, respectively) by ∓*α*. Physically, the chirality parameter *α* corresponds to the propagation distance, in wavelengths, over which the polarization vector completes a full 2*π* rotation. As discussed in Appendix A, circular birefringence induced by magneto-electric coupling due to *α* ≠ 0 results in distinct refractive indices for right- and left-circularly polarized light, namely *n*_R_ = *n* − *α* and *n*_L_ = *n* + *α*, where $n={\left(\epsilon_{r}\mu_{r}\right)}^{1/2}$ is the background refractive index.

When circular birefringence is combined with linear birefringence – arising from an anisotropic permittivity, ***ϵ***_⊥_ = diag(*ϵ*_1_, *ϵ*_2_) – the resulting medium supports coupled-wave dynamics analogous to those found in uniform Bragg gratings (see Appendix A). By contrast to conventional gratings, however, resonance now depends solely on the intrinsic parameters of the medium and the slab length, rather than on subwavelength spatial periodicity or lithographic patterning. Specifically, for a circularly and linearly birefringent medium, phase-matching leads to a “resonant” condition $\alpha =\pm \bar{n}$ . Here $\bar{n}={\left({\bar{\epsilon }}_{r}\mu_{r}\right)}^{1/2}$ denotes the average refractive index and ${\bar{\epsilon }}_{r}=\left(\epsilon_{1}+\epsilon_{2}\right)/2$ is the mean permittivity; unless stated otherwise, *μ*_*r*_ = 1.

Assuming $\bar{n}$ to be dispersionless, the detuning $\delta_{\text{R,L}}=2k_{0}\left(\bar{n}\mp \alpha \right)$ , where *k*_0_ is the free-space wavenumber, becomes wavelength-independent and determined solely by the medium parameters (a discussion of dispersion and realistic implementation using meta-media is deferred to Section 4). Furthermore, each resonant condition (centered at either $\alpha =+\bar{n}$ or $\alpha =-\bar{n}$ ) is associated with a distinct handedness. Indeed, the condition *δ*_R_ = 0 (respectively, *δ*_L_ = 0) yields a polarization-selective reflective response centered at $\alpha =\bar{n}$ (respectively, $\alpha =-\bar{n}$ ) under RCP (respectively, LCP) light excitation. In the vicinity of this regime *all* wavelengths are reflected, insofar as $\left|\alpha \left(\lambda \right)-\bar{n}\left(\lambda \right)\right|\approx 0$ , motivating the concept of a spatially uniform Bragg reflector [25]. Yet, in the absence of spatial periodicity, one may ask: how can a spectral hole arise?

It turns out that the answer lies not in the wavelength domain but in the domain of chirality. To show this, let us consider two cascaded slabs of equal length, each comprising homogeneous media exhibiting both circular and linear birefringence, as depicted in Figure 1(b). The first slab (CLB_1_), of length *L*/2, has principal permittivities (*ϵ*_1_, *ϵ*_2_) and average refractive index ${\bar{n}}_{1}={\left[\left(\epsilon_{1}+\epsilon_{2}\right)/2\right]}^{1/2}$ . The second slab (CLB_2_), also of length *L*/2, has principal permittivities (*ϵ*_2_, *ϵ*_3_) and average refractive index ${\bar{n}}_{2}={\left[\left(\epsilon_{2}+\epsilon_{3}\right)/2\right]}^{1/2}$ ; importantly *ϵ*_1_ ≠ *ϵ*_3_.

For, say, RCP excitation, each slab is characterized by a distinct set of coupled-wave theory parameters: detuning $\delta_{\text{R}}^{\left(j\right)}$ and coupling coefficient *κ* ^(*j*)^; *j* = {1, 2}. In particular, CLB_1_ has $\delta_{\text{R}}^{\left(1\right)}=2k_{0}\left({\bar{n}}_{1}-\alpha \right)$ and $\kappa^{\left(1\right)}=\left(k_{0}/2\right)\left|\epsilon_{1}^{1/2}-\epsilon_{2}^{1/2}\right|$ , whereas CLB_2_ has $\delta_{\text{R}}^{\left(2\right)}=2k_{0}\left({\bar{n}}_{2}-\alpha \right)$ and $\kappa^{\left(2\right)}=\left(k_{0}/2\right)\left|\epsilon_{2}^{1/2}-\epsilon_{3}^{1/2}\right|$ . Accordingly one can construct a transfer matrix $T_{\text{R}}^{\left(j\right)}$ for each slab. These matrices relate the electric field amplitudes at *z*_*j*_ to those at *z*_*j*−1_ via $A_{\text{R}}\left(z_{j}\right)=T_{\text{R}}^{\left(j\right)}\cdot A_{\text{R}}\left(z_{j-1}\right)$ , where $A_{\text{R}}\left(z_{j}\right)={\left(A_{\text{R}}^{+}\left(z_{j}\right),A_{\text{R}}^{-}\left(z_{j}\right)\right)}^{⊺}$ , with ^⊺^ denoting transpose and “±” indicating the direction of phase propagation.

The transfer matrix of the cascaded structure is

(1) $$T_{\text{R}}=T_{\text{R}}^{\left(2\right)}T_{\text{R}}^{\left(1\right)}=\left(\begin{array}{cc} T_{11} & T_{12}\\ T_{21} & T_{22} \end{array}\right).$$

Following the formalism of Ref. [25], which treats axial propagation in circularly and linearly birefringent media, the framework is here extended to cascaded doubly-birefringent slabs supporting spectral holes. The components of the resulting transfer matrix, whose analytic expressions are provided in Appendix A, depend on the coupled-wave parameters:

(2) $$p_{\text{R}}^{\pm ,\left(j\right)}=\cosh\left(\Delta_{\text{R}}^{\left(j\right)}L_{j}^{-}\right)\pm i\frac{\delta_{\text{R}}^{\left(j\right)}}{2\Delta_{\text{R}}^{\left(j\right)}}\sinh\left(\Delta_{\text{R}}^{\left(j\right)}L_{j}^{-}\right),$$

(3) $$q_{\text{R}}^{\pm ,\left(j\right)}=\pm i\frac{\kappa^{\left(j\right)}}{\Delta_{\text{R}}^{\left(j\right)}}\sinh\left(\Delta_{\text{R}}^{\left(j\right)}L_{j}^{-}\right),$$

with $\Delta_{\text{R}}^{\left(j\right)}={\left[{\left(\kappa^{\left(j\right)}\right)}^{2}-{\left(\delta_{\text{R}}^{\left(j\right)}/2\right)}^{2}\right]}^{1/2}$ and $L_{j}^{\pm }=z_{j}\pm z_{j-1}$ highlighting the dependence of **T**_R_ on *z*_*j*−1_ [26].

For incident RCP light, imposing the boundary condition $A_{\text{R}}^{-}\left(z=L\right)=0$ yields the right-to-right reflection and transmission coefficients: *r*_RR_ = −*T*_21_/*T*_22_ and *t*_RR_ = 1/*T*_22_, respectively. In order to isolate the reflection effects arising solely from the simultaneous presence of the two forms of birefringence, we hereafter assume that the composite finite slab is embedded in a surrounding medium of refractive index $\bar{n}$ , such that approximate index matching is achieved. Variations in transverse birefringence or in the effective slab thickness that could induce Fabry–Pérot phase shifts are presently neglected.

For a spectral hole to arise at a specific wavelength $\lambda_{0}^{\text{s.h.}}$ , two *counter*-propagating eigenmodes must interfere destructively, i.e., $r_{\text{RR}}\left(\lambda_{0}^{\text{s.h.}}\right)=0$ . This implies that *T*_21_ = 0, which upon substitution of the expressions for $p_{\text{R}}^{\pm ,\left(j\right)}$ and $q_{\text{R}}^{\pm ,\left(j\right)}$ into the formula for *T*_21_ given in Appendix A yields

(4) $$\begin{array}{ll} {\text{e}}^{\text{i}\left(\delta_{\text{R}}^{\left(2\right)}-\delta_{\text{R}}^{\left(1\right)}\right)L/2} & =-\frac{\kappa^{\left(1\right)}\tanh\left(\Delta_{\text{R}}^{\left(1\right)}L/2\right)}{\kappa^{\left(2\right)}\tanh\left(\Delta_{\text{R}}^{\left(2\right)}L/2\right)}\\ & \;\times \frac{2\Delta_{\text{R}}^{\left(2\right)}-i\delta_{\text{R}}^{\left(2\right)}\tanh\left(\Delta_{\text{R}}^{\left(2\right)}L/2\right)}{2\Delta_{\text{R}}^{\left(1\right)}+i\delta_{\text{R}}^{\left(1\right)}\tanh\left(\Delta_{\text{R}}^{\left(1\right)}L/2\right)}. \end{array}$$

It follows from Eq. (4) that the left-hand side simplifies to ${\text{e}}^{\text{i}k_{0}\left({\bar{n}}_{1}-{\bar{n}}_{2}\right)L}$ . Whence, for Eq. (4) to admit a solution for some finite value of *L*, the right-hand side must equal −1. This condition is met only if *both* media are tuned to *precisely* the same chirality parameter – a trivial scenario corresponding to unitary spectral hole transmission. To access a non-trivial regime, we perturb around this degenerate case while still retaining unit transmittance. Concretely, we consider a nearly coincident configuration in which the refractive indices of CLB_1_ and CLB_2_ differ *slightly*. Specifically, we assume that $\left|\epsilon_{2}^{1/2}-\epsilon_{1}^{1/2}\right|$ and $\left|\epsilon_{3}^{1/2}-\epsilon_{2}^{1/2}\right|$ are sufficiently small such that each slab is resonant at nearly, but not exactly, the same value of *α*. Under this near-resonant condition, Eq. (4) reduces approximately to ${\text{e}}^{\text{i}k_{0}\Delta n_{21}L_{c}}\approx -1$ , leading to the characteristic structure length for destructive interference

(5) $$L_{c}\approx \frac{\lambda_{0}}{2\Delta n_{21}},$$

where $\Delta n_{21}={\bar{n}}_{2}-{\bar{n}}_{1}$ quantifies the index mismatch.

This relation enables the design of a uniform structure supporting chiral spectral holes through an appropriate selection of the triplet (*ϵ*_1_, *ϵ*_2_, *ϵ*_3_). For ordering such that *ϵ*_1_ < *ϵ*_2_ < *ϵ*_3_ we may impose as a design strategy that *κ* ^(1)^ = *κ* ^(2)^, whence we obtain

$$2\epsilon_{2}^{1/2}=\epsilon_{1}^{1/2}+\epsilon_{3}^{1/2}.$$

Given a base permittivity *ϵ*_1_ and a perturbation *δ*_*ϵ*_ > 0, the triplet can then be cast as

(6) $$\epsilon_{1},\;\epsilon_{3}=\epsilon_{1}+\delta_{\epsilon },\;\text{and}\;\epsilon_{2}={\left[\frac{1}{2}\left(\epsilon_{1}^{1/2}+\epsilon_{3}^{1/2}\right)\right]}^{2}.$$

**Figure 2:**
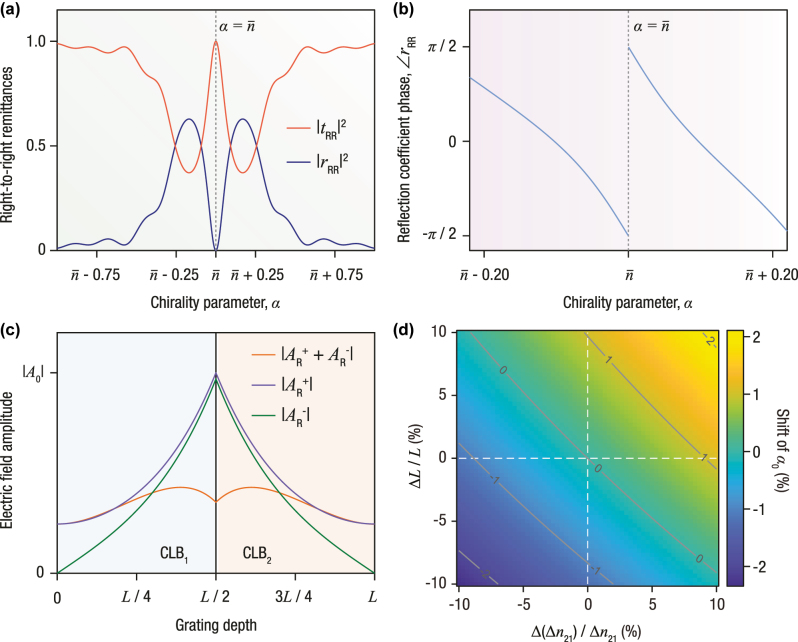
Chirality-domain spectral hole formation in cascaded circularly and linearly birefringent media. Optical response of the structure shown in Figure 1(b), when embedded in an index-matched surrounding medium of refractive index $\bar{n}$ . (a) Right-to-right intensity reflectance and transmittance (remittances) as functions of the chirality parameter *α*. The generalized interference condition of Eq. (4), combined with the characteristic slab length approximated by Eq. (5), leads to complete destructive interference at $\alpha =\bar{n}=\left({\bar{n}}_{1}+{\bar{n}}_{2}\right)/2$ , thereby generating a spectral hole in the chirality domain. By contrast to spatially periodic gratings, this mechanism requires no spatial modulation, and the resulting reflection minimum does not originate from a conventional resonance, avoiding the sharp spectral constraints typically associated with the Bragg condition of wavelength-resonant systems. The structure also exhibits polarization selectivity analogous to the circular Bragg phenomenon in structurally chiral media. (b) Phase response under the same conditions as in panel (a), featuring a sharp phase transition at the spectral hole, thus signifying the “resonant” character (in the chirality domain) of the reflection zero. (c) Spatial profiles of the forward- and backward-propagating electric field amplitudes within the structure at $\alpha =+\bar{n}$ . The fields are strongly localized near the interface between the slabs, with the backward-propagating component decaying downstream, consistent with the vanishing reflectance and near-unity transmittance observed in panel (a), without any attempt on impedance matching. (d) Two-dimensional map of the fractional shift in the chirality setting at the reflection minimum, (*α*_0_ − *α*_0,nom_)/|*α*_0,nom_|, versus fractional thickness error Δ*L*/*L* and fractional mismatch error Δ(Δ*n*_21_)/Δ*n*_21_. The reflection zero remains within a few percent of its nominal position for ±10 % variations, confirming the robustness of the interference-based mechanism. Simulation parameters follow Eq. (6), with *ϵ*_1_ = 4 and *δ*_*ϵ*_ = 2 used as representative values.

By systematically varying *δ*_*ϵ*_, one can control the separation between *ϵ*_1_ and *ϵ*_3_, thereby enabling precise numerical tuning while satisfying the constraint of Eq. (5). The intensity reflectance and transmittance spectra of such an optimized structure are shown in Figure 2(a), whereas Figure 2(b) displays the corresponding phase response. As opposed to conventional optical spectra, the abscissa here represents variations in chirality rather than wavelength.

This distinction is further evidenced in Figure 2(c), which plots the electric field amplitude distribution within the structure as a function of the grating depth. Rather than the forward-traveling wave being strongly coupled into the backward-traveling wave, the latter attains its peak at the interface of the two cascaded gratings and subsequently decays as it propagates through the remainder of the structure. By contrast to phase-shifted Bragg gratings, where this behavior is attributed to the wavelength falling within a spectral hole, the localization observed here stems from the design strategy itself, i.e., the choice of parameters satisfying Eqs. (5) and (6), upon which the chiral tuning ultimately hinges.

Insofar as the stability of the tuning condition is concerned, Eq. (5) indicates that the destructive interference condition depends primarily on the index contrast, $\Delta n_{21}={\bar{n}}_{2}-{\bar{n}}_{1}$ , and is intrinsically robust to small structural variations. A deviation of the total slab thickness from its nominal value, *L* = *L*_*c*_ + Δ*L*, introduces a phase shift Δ*ϕ* = *k*_0_Δ*n*_21_Δ*L*. However, since both modes experience nearly identical phase corrections, the spectral hole remains largely unaffected. This robustness is illustrated in Figure 2(d), where the fractional chirality shift (i.e., the displacement of the “resonance” center) associated with the reflection-zero stability is evaluated as a function of fractional thickness and refractive-index mismatch errors. In particular, simulations reveal that ±10 % variations in either *L* or *n*_1,2_ shift the reflection zero by less than 2.5 %, thereby confirming the high fabrication tolerance of the proposed interference-based mechanism.

## 3 Broadband spatial Laplacian-like differentiation

An all-optical Laplacian-like differentiator imparts a spatially uniform second-order response across the angular spectrum of an incident beam, such that the reflected (or transmitted) field closely approximates the Laplacian of the input wavefield profile in modulus. Realizing this functionality requires engineering the structure’s transfer function – that is to say, the reflection coefficient *r*_RR_ – to exhibit a quadratic dependence on the transverse wavenumbers *k*_*x*_ and *k*_*y*_. Capitalizing on the principles established in Section 2, we demonstrate in this section that Laplacian-like differentiation can be achieved via the setup of Figure 1(b) by exploiting spectral holes arising in the chirality domain.

**Figure 3:**
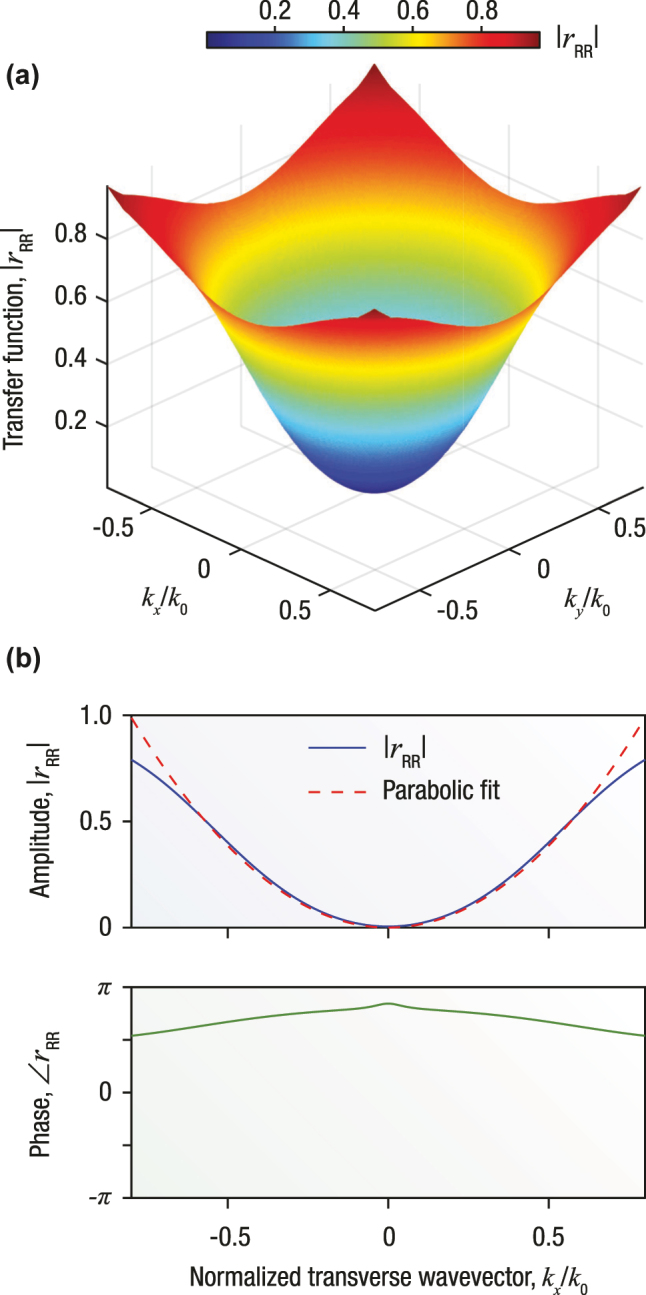
Transfer function features of the chirality-driven, all-optical Laplacian differentiator in modulus. (a) Amplitude of the reflection coefficient, |*r*_RR_|, for an incident RCP plane wave, plotted as a function of the normalized transverse wavenumbers *k*_*x*_/*k*_0_ and *k*_*y*_/*k*_0_. The resulting cone-like profile exhibits a sharp minimum at *k*_*x*_ = *k*_*y*_ = 0, consistent with the destructive interference condition *r*_RR_(*λ*, *α*) = 0 derived in Section 2. The amplitude follows a symmetric quadratic dependence, signature of Laplacian-type behaviour. (b) Amplitude (top panel) and phase (bottom panel) of the reflection coefficient as functions of *k*_*x*_/*k*_0_. The amplitude curve is fitted with a parabola (dashed red line), whose curvature determines the proportionality constant *C* in Eq. (7). The phase response varies smoothly across the reflection minimum without discontinuities, indicating a high-fidelity implementation of a Laplacian operator in modulus acting on the angular spectrum of the incident field.

In principle Laplacian differentiation emerges from the steep parabolic dependence of the reflection coefficient in the vicinity of its zero. The condition of destructive interference, *r*_RR_(*λ*, *α*) = 0, defines a spectrally sharp reflection minimum wherein both the magnitude |*r*_RR_| and the phase $∠\left(r_{\text{RR}}\right)$ vary rapidly yet in a symmetric fashion. For an incident plane wave, Figure 3(a) depicts the three-dimensional surface of the reflection coefficient amplitude as a function of the normalized transverse wavenumbers *k*_*x*_/*k*_0_ and *k*_*y*_/*k*_0_. The resulting parabolic surface profile, proportional to $k_{x}^{2}+k_{y}^{2}$ , clearly exhibits the hallmark characteristic of a Laplacian operator in modulus across the transverse spatial frequency domain. Focusing on the *k*_*x*_-axis, the top panel of Figure 3(b) shows that the amplitude of the reflection coefficient is approximately symmetric and quadratic in *k*_*x*_, overlaid with a parabolic fit, where the remarkable agreement even for high values of *k*_*x*_ underscores both the quality of the engineered spectral hole and the overall performance of the Laplacian-like operator. The corresponding phase response, shown in the bottom panel of Figure 3(b), reveals that near the zero-reflection point, the phase evolves continuously and without discontinuity. Crucially, while the slab length sets a design wavelength, the formation of the spectral hole is governed by material parameter tuning rather than a conventional resonance condition. In this context *high*-quality pertains to the *exact* fulfillment of the condition $r_{\text{RR}}\left(\lambda ,\alpha =\pm \bar{n}\right)=0$ across the relevant (i.e., operational) spectral range.

Mathematically, such a Laplacian-like optical response can be cast in terms of an effective operator acting on the input field. Indeed, let us consider a paraxial beam with transverse profile *E*_*i*_(*x*, *y*) incident on the structure. The angular spectrum of the reflected field is

$$E_{r}\left(x,y\right)=\overset{\infty }{\underset{-\infty }{\iint }}r_{\text{RR}}\left(k_{x},k_{y}\right)\;{\tilde{E}}_{i}\left(k_{x},k_{y}\right)\;{\text{e}}^{\text{i}\left(k_{x}x+k_{y}y\right)}\;dk_{x}\;dk_{y},$$

where ${\tilde{E}}_{i}\left(k_{x},k_{y}\right)$ is the 2D Fourier transform of *E*_*i*_(*x*, *y*). If the transfer function satisfies $r_{\text{RR}}\left(k_{x},k_{y}\right)\propto -\left(k_{x}^{2}+k_{y}^{2}\right)$ , then the reflected field approximates the Laplacian of the input profile,

(7) $$E_{r}\left(x,y\right)\approx -C\;\nabla^{2}E_{i}\left(x,y\right),$$

with *C* being a proportionality constant determined by the curvature of *r*_RR_ in the vicinity of the spectral hole.

Since tuning is achieved through parameter matching rather than conventional Bragg resonance, the quadratic approximation of *r*_RR_(*k*_*x*_, *k*_*y*_) near the reflection minimum does not rely on a narrowband resonant condition. Instead, it remains valid over a finite spectral region around the design wavelength determined by the slab length. Although the range is not inherently wavelength-independent, it is not constrained by the sharp spectral selectivity typical of resonant structures. If the dispersion of *α* and $\bar{n}$ is closely matched, the detuning remains small across a finite bandwidth, preserving the Laplacian-like response within practical limits. The curvature of the phase response near the reflection zero can be adjusted through the relative permittivity contrast, specified by the design parameter *δ*_*ϵ*_ in Eq. (6), offering additional control over the angular response of the differentiator.

Furthermore, a salient feature of our mechanism lies in its intrinsic polarization selectivity. Indeed, the transfer function *r*_RR_(*k*_*x*_, *k*_*y*_) is inherently handedness-dependent: for a given sense of optical rotation, only one circular polarization state (RCP or LCP) experiences the chiral stopband, while the opposite handedness propagates unaffected, subject only to absorption. This selectivity arises from the symmetry breaking induced by the chirality parameter *α*, which couples asymmetrically to the two orthogonal states. Hence, the structure performs Laplacian differentiation in modulus exclusively on the selected polarization component, enabling polarization-resolved spatial processing. If a linearly polarized beam – comprising equal amounts of RCP and LCP light – is incident, only the component aligned with the chosen chirality sign is differentiated in the corresponding channel.

Reversing the order of the two slabs interchanges the handedness of circular birefringence and yields an equivalent Laplacian response up to a mirror inversion of the output field, leaving the spectral-hole position and its parabolic *k*_⊥_ dependence essentially unchanged, while merely reversing the internal phase of *r*_RR_ and the direction of field localization. This mirror equivalence highlights the system’s reciprocal nature: in a non-reciprocal counterpart, such an inversion would no longer produce an equivalent response but rather a distinct, direction-dependent behaviour. Building upon the framework introduced in Ref. [27], this interference mechanism could be extended to magneto-optic or gyrotropic heterostructures to realize asymmetric (i.e., non-reciprocal) spatial differentiation, wherein forward and backward propagation elicit distinct responses. In such media, direction-dependent magnetoelectric coupling enables selective enhancement or suppression of spatial features, extending spectral-hole interference to non-reciprocal regimes. Recent demonstrations of strong non-reciprocity in gyrotropic and hybrid ferrite–ferroelectric multilayers [28], [29], [30] confirm that the material parameters needed for the Bragg-like interference central to our design are already experimentally accessible, paving the way for direction-selective analogue differentiation and dynamic optical control.

**Figure 4:**
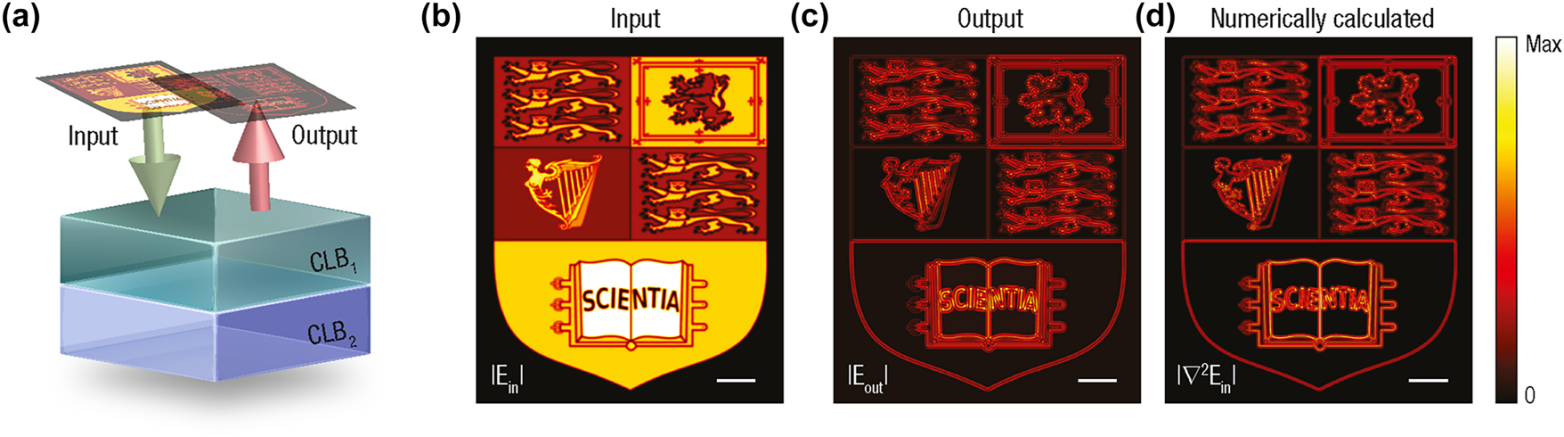
Chirality-driven, all-optical Laplacian operator in modulus enabling broadband, high-fidelity edge detection. (a) Schematic of the proposed Laplacian-like differentiator: for the configuration displayed in Figure 1(b), when surrounded by an isotropic medium of refractive index $\bar{n}$ , the reflected signal corresponds to the modulus of the Laplacian of the electric field profile of the incident lightwave. (b) Normalized intensity profile of the input beam, |*E*_in_|, used to excite the structure. (c) Simulated amplitude of the reflected field, |*E*_out_|, based on the transfer function illustrated in Figure 3. The output accurately reproduces the high-contrast features expected from Laplacian-based edge detection. (d) Numerically calculated magnitude of the Laplacian of the input profile, |∇^2^ *E*_in_|, shown for comparison. The proposed scheme accurately reconstructs the edges and fine details of the Imperial College crest, in excellent agreement with those obtained from the analytical Laplacian [cf. panels (c) and (d)]. The scale bars in panels (b)–(d) correspond to 20 × 2*π*/*k*_0_.

To illustrate the practical capabilities of the proposed differentiating scheme, Figure 4 presents simulations using the Imperial College crest as the input field profile [see Figure 4(b)]. The structure used in Figure 4(a) corresponds to the configuration in Figure 1(b), designed to produce a high-quality reflection zero at the target chirality parameter *α*. Upon illumination, the reflected field reconstructs the Laplacian of the input image in modulus, highlighting edges and fine features with high fidelity. The output amplitude closely reproduces the numerically simulated one across the entire field of view [cf. panels (c) and (d) in Figure 4], confirming the precision of chirality-domain tuning. Further simulations have confirmed that such a behaviour remains stable under variations in slab thickness and the chirality parameter. The output edge-enhanced images demonstrate that high-contrast, polarization-selective spatial processing can be realized with the proposed configuration, providing a scalable alternative to conventional grating- or metasurface-based edge detectors.

**Figure 5:**
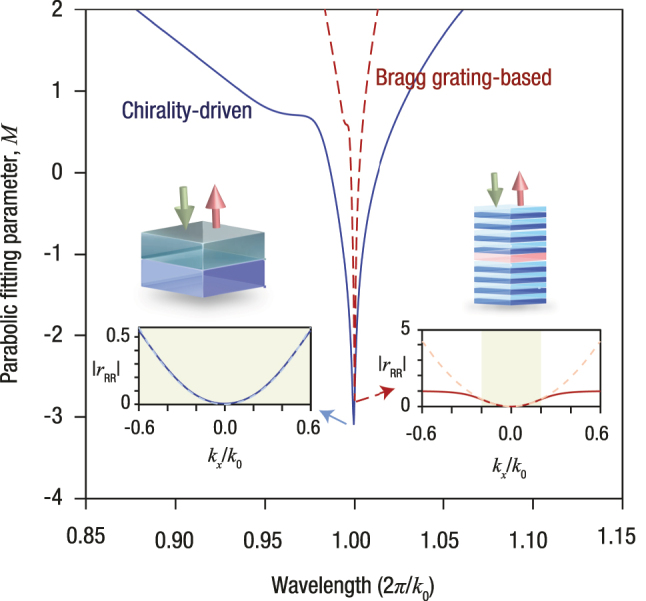
Comparison of operational bandwidths between the proposed chirality-driven and traditional Bragg grating-based differentiators. Parabolic fitting parameter *M* is plotted as a function of normalized wavelength for both the chirality-driven (blue solid line) and Bragg grating-based (red dashed line) spatial differentiators. Insets display the reflection coefficient magnitude as a function of normalized transverse wavenumber (*k*_*x*_/*k*_0_) for each case at the operational wavelength 2*π*/*k*_0_, with solid curves representing the simulated data and dashed curves indicating the corresponding parabolic fits. The chirality-driven device exhibits a well-matched quadratic response over a broad angular range, *k*_*x*_/*k*_0_ ∈ [−0.6, 0.6], whereas the 17-layer Bragg grating design of Ref. [17] maintains parabolic behaviour only within the narrower interval *k*_*x*_/*k*_0_ ∈ [−0.2, 0.2]. As elaborated in Appendix B, lower values of *M* correspond to a better parabolic approximation of the transfer function. The dispersive material properties for the chirality-driven differentiator are given in Appendix C.

Finally, in Figure 5, we benchmark the performance of the chirality-driven spatial differentiator against that of a conventional Bragg grating device, based on the 17-layer configuration proposed in Ref. [17]. Since dispersion is inherently linked to negative refraction, we incorporate a physically consistent comparison by modeling the chirality-based structure using Lorentz-type dispersion for both the permittivity and chirality parameters [see Eqs. (14a)–(14c) in Appendix C]. When negative refraction is present, dispersion is inevitable, since any stable system must possess a positive electromagnetic energy density. This requirement can be established by equating the energy flow, determined from the group velocity $v_{g}={\left|\text{d}k/\text{d}\omega \right|}^{-1}$ , with the Poynting vector. Considering the forward-propagating RCP mode [cf. $k_{\text{R}}^{+}=k_{0}\left(\bar{n}-\alpha \right)$ in Eq. (11)], this condition leads to

$$u_{g}>0\Rightarrow \frac{\text{d}}{\text{d}\omega }\left[\bar{n}\left(\omega \right)-\alpha \left(\omega \right)\right]>-\frac{\bar{n}\left(\omega \right)-\alpha \left(\omega \right)}{\omega }.$$

This inequality, together with the dispersive models of Appendix C, can be used to estimate bounds on bandwidth.

The bound implied above reflects the practical limits imposed by dispersion: although intrinsically non-Bragg, the operational bandwidth is constrained by the wavelength dependence of the chirality *α*(*λ*) and the refractive indices *n*_1,2_(*λ*) in Eq. (5). For existing chiral metamaterials [22], [31], the attainable fractional bandwidth lies between 10^−2^ and 10^−1^, consistent with the ∼1 % value predicted by our model and comparable to broadband differentiators based on dielectric metasurfaces [4], [32], [33], cholesteric-liquid-crystal architectures [34], [35], [36], and spin–orbit-coupled crystals [37]. The distinction of our approach lies not in the absolute bandwidth but in its *origin*: the response arises from interference between contra-handed, counter-propagating circular eigenwaves governed by *α*, *n*_1,2_, and *L*_*c*_, rather than by a fixed lattice period, and is moreover inherently polarization-selective. Although dispersion limits the usable range, the same interference principle can be extended to other spectral regions through appropriate dispersion engineering. Parameter control – assisted, e.g., by orthogonal-wire inclusions that tune the plasma frequency [38] – enables operation without necessarily rescaling the meta-atom geometry, while further tunability may be achieved via dynamic modulation of *α* or by varying the inclination angle between the optical axis and the propagation direction [39].

At normal incidence, the circular eigenmodes are decoupled. At oblique incidence, although the bulk modes within each isotropic chiral slab remain circular, TE and TM fields hybridize at the interfaces, introducing angle-dependent propagation constants and two additional degrees of freedom: incidence angle and polarization. Generalizing the destructive-interference condition to e^iΔ*β*(*θ*)*L*^ ≈ −1, where Δ*β*(*θ*) denotes the angle-dependent mismatch between the slabs, preserves the filtering behaviour while rendering the response tunable through both angle and input polarization. In a related controllability scheme, Ref. [39] shows that off-axis propagation relaxes the tuning condition, enabling ultra-narrow spectral holes with weaker chirality and allowing precise control of the resonance location and corresponding bandwidth via the angle formed between the optical axis and the direction of wave propagation. Moreover, under near-total internal reflection, angle–polarization coupling can yield exceptionally sharp angular filtering [40], [41]. Our cascaded model can be extended to capture these effects by solving for spin-coupled modes, in which TE/TM hybridization and circular birefringence coexist. The resulting transfer function remains locally quadratic around the operating point (*k*_*x*0_, *k*_*y*0_), corresponding to a direction-dependent second-order differentiation that preserves the Laplacian-like behaviour at normal incidence while providing additional pathways for tunability and angular selectivity.

Using the same parameters as in Ref. [17], Figure 5 plots the parabolic fitting metric *M*, defined in Eq. (13) of Appendix B, as a function of normalized transverse wavenumber *k*_*x*_/*k*_0_. Evidently, the chirality-driven structure maintains a more accurate parabolic response over a broader angular range. Indeed, the normalized spectral bandwidth over which *M* < −1 extends to 0.010 within *k*_*x*_/*k*_0_ ∈ [−0.6, 0.6] for the chirality-driven design, compared to only 0.004 within *k*_*x*_/*k*_0_ ∈ [−0.2, 0.2] for the Bragg-based device – over a factor of two improvement in bandwidth, sustained across a threefold larger angular span.

This distinction arises from the different physical mechanisms underpinning the two designs. In Bragg gratings, the reflection minimum emerges from constructive interference in a periodic index profile, and thus obeys a resonance condition. This introduces an inherent trade-off: increasing the number of grating layers deepens the reflection minimum but narrows the bandwidth over which a clean quadratic response is preserved. By contrast, the chirality-based structure does not rely on a periodic modulation or resonance. Its response is engineered through parameter tuning in a uniform, non-periodic slab with a controlled dispersive response. This avoids the bandwidth-versus-reflectivity trade-off of conventional Bragg gratings and enables a broader spectral range without compromising the quality of the Laplacian-like response – even in the presence of realistic material dispersion.

## 4 Meta-media implementation

At the core of the proposed mechanism lies a Bragg-like resonance in the chirality domain, governed by stopbands that emerge from parameter matching rather than conventional wavelength matching. As a result, the spectral range over which unity reflectance is achieved is centered around the design wavelength set by the slab length, but it is not governed by a wavelength/frequency domain resonance condition and is therefore not limited by the narrow spectral constraints typical of resonant systems. Although intrinsic material dispersion in the refractive index and chirality, $\bar{n}\left(\lambda \right)$ and *α*(*λ*), limits the bandwidth over which polarization-selective reflection can occur, these effects can be mitigated through appropriate material design. For example, the archetypal metamaterial of Ref. [38], consisting of a host medium embedded with uniformly distributed metallic Mie resonators, was shown in Ref. [25] to support robust stopbands even at low free-electron densities. Although moderate spectral shifts occur, fine-tuning the electron density stabilizes these stopbands *near* $\alpha =\pm \bar{n}$ .

Moreover, recent advances in nanofabrication have enabled remarkably large chiral responses across a broad range of the electromagnetic spectrum. At optical frequencies, for instance, Ref. [42] demonstrated *α* ≈ 0.15 at 540 nm using gammadion geometries. Dielectric metasurfaces have pushed these values significantly higher: optimized vertically offset dielectric bar pairs achieved *α* ≈ 3 over 596–604 nm [31], while all-dielectric toroidal dipole architectures demonstrated *α* ≈ 4.8 between 975 and 995 nm [22]. At longer wavelengths, Ref. [21] reported *α* ≈ 2.45 at 0.27 mm in a three-dimensional gold-based chiral meta-medium, and in the centimeter regime, Ref. [43] observed *α* values between −0.62 and −2.65 at 5.08–6.52 cm using a four-layer rosette-based metamaterial.

A key challenge in designing a meta-medium capable of supporting such a spectral response lies in the distinct dispersive behaviour of permittivity and chirality. Indeed, permittivity typically exhibits Lorentzian dispersion shaped by electronic or molecular resonances, whereas chirality – originating from magnetoelectric coupling – is sharply peaked near resonance and decays rapidly off-resonance [cf. Eqs. (14a) and (14c) in Appendix C]. This difference complicates simultaneous engineering of both parameters over a common frequency band. Structural approaches, however, can mitigate this disparity. Indeed, arrays of interconnected helices support continuous current pathways, hence broadening Lorentz resonances and enabling broadband optical activity with reduced dispersion [44]. Bilayer metasurfaces pairing chiral elements with complementary structures yield spectrally uniform optical rotation [45], whilst recently developed planar metasurfaces sustain broadband chirality [46].

Dynamic control of chirality has only recently become experimentally viable, driven by progress in advanced 3D fabrication. In the near-infrared, triple-helical platinum nanowire metamaterials achieved tunable chirality in the range *α* ∈ (0.024, 0.032) across 750–1000 nm [47]. At longer wavelengths, electromechanical pneumatic force produced tunability over *α* ∈ (−1.59, 0.43) across 0.25–0.37 mm [48], and piezoelectrically actuated kirigami modulators achieved *α* ∈ (0, 1.71) from 0.37 to 1.5 mm [49]. Electric-field-controlled metasurfaces reached *α* ∈ (−0.37, 0.67) over 1.52–1.75 μm [50], while conductivity-controlled designs demonstrated tunability across *α* ∈ (−0.82, 2.89) from 0.4 to 5 mm [23].

Nonreciprocal bi-anisotropic media offer further opportunities for dynamic control. Recent advances in nonlocal nonlinear metasurfaces [51] and space-time-modulated media [52] suggest that substantial enhancement of nonreciprocal responses is now within reach. Moreover, certain Tellegen media – characterized by purely real magnetoelectric coupling several orders of magnitude stronger than those found in Nature [53] – can reproduce key features of bandgap formation, thus enabling tunable nonreciprocal modulation through, for example, polarization currents.

Metamaterial architectures based on rosette, cross-wire, split-ring, and spiral geometries routinely achieve fractional bandwidths that exceed those of cholesteric liquid crystals (typically around 12 %), with reported values surpassing 30 % (consult Table 1 in Ref. [39] and references therein). This degree of spectral tunability underscores the potential of meta-media to deliver an “optical advantage,” enabling passive, energy-efficient operation with extensive spatial and spectral parallelism [54]. Such capabilities are particularly relevant given the increasing spatial complexity of optical computing, where recent scaling laws relate the physical dimensions of photonic systems to the computational tasks they perform [55].

## 5 Conclusions

This work introduces a broadband, polarization-sensitive all-optical Laplacian operator in modulus, realized via engineered “spectral holes” in cascaded, doubly birefringent uniform slabs with slightly mismatched refractive indices. Through coupled-wave theory analyses we have demonstrated that tuning of material parameters enables the formation of high-quality spectral holes in the chirality domain, without requiring spatial periodicity or resonance, both of which commonly limit conventional designs. Indeed, by contrast to traditional Bragg-based scattering mechanisms, where periodicity constrains bandwidth and imposes stringent fabrication tolerances, the different physical mechanism of our structure enables operation without spatial periodicity or resonant filtering, thereby relaxing fabrication constraints. Moreover, chirality introduces the additional degree of freedom necessary to enable polarization-selective Laplacian differentiation in modulus – a functionality that, to the best of our knowledge, has remained largely unexplored. The performance of the proposed design is further supported by its near-ideal parabolic transfer function, maintained over a broad transverse-wavevector range, thereby ensuring robust spatial differentiation across a wider angular band than attainable with conventional Bragg-based architectures.

The proposed platform leverages recent advances in meta-optics, particularly in bi-anisotropic metamaterials capable of supporting giant and tunable chirality over broad spectral ranges. These developments not only enable practical realizations of the proposed edge-detection scheme but also open new directions for reconfigurable contrast enhancement and pattern recognition.

While edge detection serves as a canonical illustration of the Laplacian-like response, the cascaded-chirality framework naturally accommodates related spatial operations within the same physical principle. Directional differentiation can be realized by introducing a controlled asymmetry between the two slabs, and the intrinsic polarization selectivity enables spin-resolved image processing. Similar functionalities have been proposed for Bragg-based differentiators in the context of edge enhancement and mode conversion (see, e.g., Refs. [17], [18]), yet the present approach achieves a substantially higher degree of parabolicity around the reflection minimum than typical Bragg gratings, allowing faithful Laplacian operation over a broader spatial-frequency range. This higher-order accuracy is crucial for preserving edge fidelity while maintaining background suppression. In addition, the operating point depends mainly on the average material parameters and slab thicknesses, avoiding the need for precise spectral optimization and offering strong tolerance to fabrication or dimensional variations. This simplicity in implementation makes the framework adaptable to different spatial operator designs without increasing structural complexity. Such extensions, achievable through modest tuning of the chirality contrast or slab thickness, remain consistent with the underlying interference mechanism. Furthermore, optical differentiators of this kind are increasingly employed as physical pre-processors in machine-vision and neural-network pipelines [32], providing real-time, low-power edge enhancement prior to digital analysis. These considerations underscore the broader potential of the proposed approach for compact, reconfigurable analogue image processing beyond conventional, polarization-independent designs.

Together, these results outline a promising route towards compact, broadband, and reconfigurable platforms for all-optical analog computing. As capabilities in nanofabrication, chirality control, or even synthetic bi-anisotropy continue to mature, such metamaterials may enable not only the edge-detection device introduced here, but also multifunctional photonic components (e.g., modulators and ultra-sensitive sensors) thereby broadening the scope of integrated optics.

## Figures and Tables

**Figure 6:**
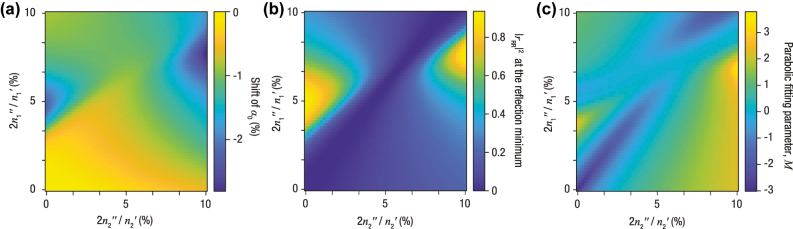
Loss dependence of the reflection zero and parabolic curvature. Two-dimensional sweeps over independent slab losses (reported as loss tangents $2n_{1}^{\prime \prime }/n_{1}^{\prime }$ for CLB_1_ and $2n_{2}^{\prime \prime }/n_{2}^{\prime }$ for CLB_2_) at normal incidence, with fixed geometry and a sweep over *α*. (a) Shift of the chirality setting at the reflection minimum, expressed as a percentage relative to the no-loss baseline *α*_0_, with local tracking to stay on the same dip. (b) Reflectance |*r*_RR_|^2^ evaluated at the tracked minimum. (c) Parabolic fitting parameter *M* computed at the tracked reflection dip over *k*_*x*_/*k*_0_ ∈ [−0.6, 0.6]. Loss tangents are varied from 0 to 10 % along both axes (horizontal: CLB_2_; vertical: CLB_1_).

**Figure 7:**
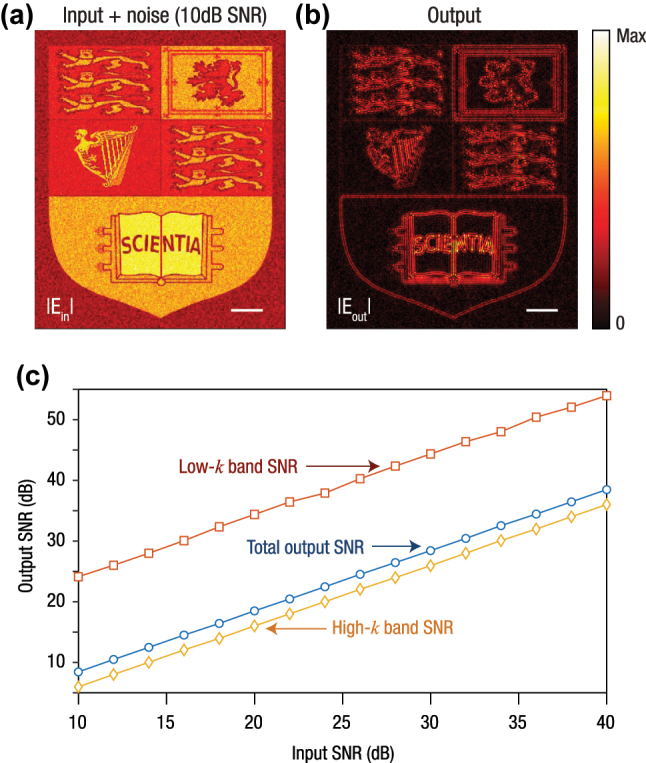
Noise propagation through the optical differentiator. (a) Input amplitude image (having 10 dB SNR) with added zero-mean white Gaussian noise on the amplitude. (b) Device output magnitude |*E*_*r*_| after filtering with the reflection transfer *R*(**k**_⊥_). (c) Output SNR summary: image-domain SNR (blue) and spectral SNR in low-*k* (0–0.15 *k*_0_, red) and high-*k* (0.30–0.60 *k*_0_, yellow) bands, evaluated with the same numerical NA cap *k* ≤ 0.6*k*_0_. The scale bars in panels (a) and (b) correspond to 20 × 2*π*/*k*_0_.

## Appendix A: Cascaded circularly and linearly birefringent media

Let **E** and **B** denote the phasors of the fundamental electric and magnetic fields, respectively, and let **D** and **H** represent their corresponding induced excitations. We may then define the auxiliary fields **b** = *c*_0_ **B**, $d=\epsilon_{0}^{-1}D$ , and **h** = *η*_0_ **H**, all possessing the same units as the electric field. Here *ϵ*_0_, *μ*_0_, $\eta_{0}={\left(\mu_{0}/\epsilon_{0}\right)}^{1/2}$ , and $c_{0}={\left(\epsilon_{0}\mu_{0}\right)}^{-1/2}$ denote the vacuum permittivity, permeability, impedance, and phase velocity of light, respectively.

For a reciprocal bi-isotropic medium, Tellegen’s constitutive relations in the frequency domain read

$$\left(\begin{array}{c} d\\ b \end{array}\right)=\left(\begin{array}{cc} \epsilon_{r} & i\alpha \\ -i\alpha & \mu_{r} \end{array}\right)\left(\begin{array}{c} E\\ h \end{array}\right),$$

with symbols as defined in the main text. For monochromatic excitation with an implicit e^−i*ωt*^ convention, Maxwell’s macroscopic source-free curl relations become

(8a)
$$\nabla \times E=k_{0}\alpha E+ik_{0}\mu_{r}h,$$

(8b)
$$\nabla \times h=-ik_{0}\epsilon_{r}E+k_{0}\alpha h.$$

Assuming plane-wave propagation along the *z*-axis, the Bohren decomposition [56] decouples the system of Eqs. (8a) and (8b) into two subsystems with orthogonal circular eigenstates. For weak chirality, |*α*| < Re(*n*), the forward-propagating RCP and LCP eigenmodes are

$$\frac{A_{\text{R}}^{+}}{\sqrt{2}}{\text{e}}^{\text{i}k_{0}\left(n-\alpha \right)z}\left(\begin{array}{c} 1\\ -i \end{array}\right)\;\text{and}\;\frac{A_{\text{L}}^{+}}{\sqrt{2}}{\text{e}}^{\text{i}k_{0}\left(n+\alpha \right)z}\left(\begin{array}{c} 1\\ i \end{array}\right),$$

respectively, whilst the backward-propagating modes are

$$\frac{A_{\text{R}}^{-}}{\sqrt{2}}{\text{e}}^{-\text{i}k_{0}\left(n+\alpha \right)z}\left(\begin{array}{c} 1\\ i \end{array}\right)\;\text{and}\;\frac{A_{\text{L}}^{-}}{\sqrt{2}}{\text{e}}^{-\text{i}k_{0}\left(n-\alpha \right)z}\left(\begin{array}{c} 1\\ -i \end{array}\right),$$

with $A_{\text{R}}^{\pm }$ , $A_{\text{L}}^{\pm }$ being constant amplitudes. For giant-chirality, |*α*| ≥ Re(*n*), the handedness and direction of phase propagation of counter-propagating modes are interchanged, as two of the modes refract negatively [57].

Linear birefringence is subsequently introduced by a relative permittivity tensor. In suitable coordinates chosen to diagonalize the latter, its transverse components read ***ϵ***_⊥_ = diag(*ϵ*_1_, *ϵ*_2_). Replacing the scalar permittivity *ϵ*_r_ with the ***ϵ***_⊥_ tensor in Eqs. (8a) and (8b), the chiral Helmholtz equation for the transverse electric field reads

(10)
$$\frac{{\text{d}}^{2}E_{⊥}}{\text{d}z^{2}}+2\alpha k_{0}\frac{\text{d}}{\text{d}z}\left(\hat{z}\times E_{⊥}\right)+k_{0}^{2}\left(\epsilon_{⊥}\mu_{\text{r}}-\alpha^{2}\right)E_{⊥}=0,$$

describing axial propagation in a CLB medium.

The coupled-wave relations follow the formalism developed in Ref. [25] for axial propagation in circularly and linearly birefringent media, here extended to cascaded doubly-birefringent slabs supporting the interference condition of Eq. (5). This can be achieved by adopting an ansatz that treats linear birefringence as a perturbation to the purely circularly birefringent eigenmodes, viz.,

(11)
$$\begin{array}{ll} E_{⊥} & =\left[\frac{A_{\text{L}}^{+}}{\sqrt{2}}{\text{e}}^{\text{i}k_{0}\left(\bar{n}+\alpha \right)z}+\frac{A_{\text{R}}^{-}}{\sqrt{2}}{\text{e}}^{-\text{i}k_{0}\left(\bar{n}-\alpha \right)z}\right]\left(\begin{array}{c} 1\\ i \end{array}\right)\\ & \;+\left[\frac{A_{\text{L}}^{-}}{\sqrt{2}}{\text{e}}^{-\text{i}k_{0}\left(\bar{n}+\alpha \right)z}+\frac{A_{\text{R}}^{+}}{\sqrt{2}}{\text{e}}^{\text{i}k_{0}\left(\bar{n}-\alpha \right)z}\right]\left(\begin{array}{c} 1\\ -i \end{array}\right). \end{array}$$

Substituting Eqs. (11) into (10), the slowly varying envelope approximation leads to two sets of coupled-wave equations. Considering RCP light excitation we write [25]

(12)
$$\frac{\text{d}}{\text{d}z}\left(\begin{array}{c} A_{\text{R}}^{+}\\ A_{\text{R}}^{-} \end{array}\right)=\left(\begin{array}{cc} 0 & i\kappa {\text{e}}^{-\text{i}\delta_{\text{R}}z}\\ -i\kappa {\text{e}}^{\text{i}\delta_{\text{R}}z} & 0 \end{array}\right)\left(\begin{array}{c} A_{\text{R}}^{+}\\ A_{\text{R}}^{-} \end{array}\right).$$

For the *j*-th slab of a CLB medium, Eq. (12) permits analytic solutions yielding a transfer matrix

$$T_{\text{R}}^{\left(j\right)}=\left(\begin{array}{cc} {\text{e}}^{-\text{i}\delta_{\text{R}}^{\left(j\right)}L_{j}^{-}/2}p_{\text{R}}^{+,\left(j\right)} & {\text{e}}^{-\text{i}\delta_{\text{R}}^{\left(j\right)}L_{j}^{+}/2}q_{\text{R}}^{+,\left(j\right)}\\ {\text{e}}^{\text{i}\delta_{\text{R}}^{\left(j\right)}L_{j}^{+}/2}q_{\text{R}}^{-,\left(j\right)} & {\text{e}}^{\text{i}\delta_{\text{R}}^{\left(j\right)}L_{j}^{-}/2}p_{\text{R}}^{-,\left(j\right)} \end{array}\right),$$

where the definitions of $p_{\text{R}}^{\pm ,\left(j\right)}$ and $q_{\text{R}}^{\pm ,\left(j\right)}$ are given by Eqs. (2) and (3) in the main text.

For the cascaded geometry of two equal-length slabs of CLB, the total transfer matrix $T_{\text{R}}=T_{\text{R}}^{\left(2\right)}T_{\text{R}}^{\left(1\right)}$ , whose representative form is given in Eq. (1), has components:

$$\begin{array}{ll} T_{11} & ={\text{e}}^{-\text{i}\delta_{\text{R}}^{\left(2\right)}L/4}p_{\text{R}}^{+,\left(2\right)}{\text{e}}^{-\text{i}\delta_{\text{R}}^{\left(1\right)}L/4}p_{\text{R}}^{+,\left(1\right)}+{\text{e}}^{-\text{i}3\delta_{\text{R}}^{\left(2\right)}L/4}q_{\text{R}}^{+,\left(2\right)}{\text{e}}^{\text{i}\delta_{\text{R}}^{\left(1\right)}L/4}q_{\text{R}}^{-,\left(1\right)},\\ T_{12} & ={\text{e}}^{-\text{i}\delta_{\text{R}}^{\left(2\right)}L/4}p_{\text{R}}^{+,\left(2\right)}{\text{e}}^{-\text{i}\delta_{\text{R}}^{\left(1\right)}L/4}q_{\text{R}}^{+,\left(1\right)}+{\text{e}}^{-\text{i}3\delta_{\text{R}}^{\left(2\right)}L/4}q_{\text{R}}^{+,\left(2\right)}{\text{e}}^{\text{i}\delta_{\text{R}}^{\left(1\right)}L/4}p_{\text{R}}^{-,\left(1\right)},\\ T_{21} & ={\text{e}}^{\text{i}3\delta_{\text{R}}^{\left(2\right)}L/4}q_{\text{R}}^{-,\left(2\right)}{\text{e}}^{-\text{i}\delta_{\text{R}}^{\left(1\right)}L/4}p_{\text{R}}^{+,\left(1\right)}+{\text{e}}^{\text{i}\delta_{\text{R}}^{\left(2\right)}L/4}p_{\text{R}}^{-,\left(2\right)}{\text{e}}^{\text{i}\delta_{\text{R}}^{\left(1\right)}L/4}q_{\text{R}}^{-,\left(1\right)},\\ T_{22} & ={\text{e}}^{\text{i}3\delta_{\text{R}}^{\left(2\right)}L/4}q_{\text{R}}^{-,\left(2\right)}{\text{e}}^{-\text{i}\delta_{\text{R}}^{\left(1\right)}L/4}q_{\text{R}}^{+,\left(1\right)}+{\text{e}}^{\text{i}\delta_{\text{R}}^{\left(2\right)}L/4}p_{\text{R}}^{-,\left(2\right)}{\text{e}}^{\text{i}\delta_{\text{R}}^{\left(1\right)}L/4}p_{\text{R}}^{-,\left(1\right)}. \end{array}$$

## Appendix B: Parabolic-fitting parameter *M*

Here we quantify how closely the transfer-function magnitude |*R*(*k*_*x*_)| follows an ideal quadratic $a\;k_{x}^{2}$ over the range |*k*_*x*_| ≤ *k*_*x*, max_; *a* is a free parameter. We begin by defining the residual variance

$$S_{\text{res}}=\overset{+k_{x,\max}}{\underset{-k_{x,\max}}{\int }}{\left[|R\left(k_{x}\right)|-a\;k_{x}^{2}\right]}^{2}\;dk_{x},$$

and the total variance

$$S_{\text{tot}}=\overset{+k_{x,\max}}{\underset{-k_{x,\max}}{\int }}{\left[|R\left(k_{x}\right)|-\bar{\left|R\right|}\right]}^{2}\;dk_{x},$$

where

$$\bar{\left|R\right|}=\frac{1}{2\;k_{x,\max}}\overset{+k_{x,\max}}{\underset{-k_{x,\max}}{\int }}|R\left(k_{x}\right)|\;dk_{x}$$

is the mean magnitude in that interval. The coefficient of determination is then

$$\rho^{2}=1-\frac{S_{\text{res}}}{S_{\text{tot}}},$$

so that the parabolic-fitting parameter *M* is defined by

(13)
$$M=\log_{10}\left(1-\rho^{2}\right)=\log_{10}\left(\frac{S_{\text{res}}}{S_{\text{tot}}}\right).$$

A more negative value of *M* indicates a closer match to the ideal quadratic dependence. The choice of *k*_*x*, max_ determines the maximum normalized transverse wavenumber over which the fit is performed.

## Appendix C: Lorentzian material dispersion

The broadband response in Figure 5 is computed using a more realistic material model in which both the permittivity tensor and the chirality parameter follow a common Lorentzian dispersion, namely

(14a)
$$\epsilon_{xx}\left(\omega \right)=\epsilon_{\infty }+\frac{F_{\epsilon }}{\omega_{0}^{2}-\omega^{2}-i\Gamma \omega },$$

(14b)
$$\epsilon_{yy}\left(\omega \right)=\epsilon_{\infty }+\frac{F_{\epsilon }^{\prime }}{\omega_{0}^{2}-\omega^{2}-i\Gamma \omega },$$

(14c)
$$\alpha \left(\omega \right)=\frac{F_{\alpha }\;\omega \left(\omega_{0}^{2}-\omega^{2}\right)}{{\left(\omega_{0}^{2}-\omega^{2}\right)}^{2}+{\left(\Gamma \omega \right)}^{2}}.$$

Here *ω*_0_ = 2*πc*_0_/*λ*_0,*r*_ is the resonance frequency, Γ = 0 is the damping rate, and *ϵ*_∞_ = 1 is the high-frequency limit. In Figure 5 the resonance wavelength is chosen as *λ*_0,*r*_ = 2*λ*_0_.

At the design wavelength *λ*_0_, the real parts of the permittivities are constrained to

$$\begin{array}{ll} \text{Slab }1\text{:} & \;\text{Re}\left\{\epsilon_{xx}\right\}=4.0,\\ \text{Slab }2\text{:} & \;\text{Re}\left\{\epsilon_{xx}\right\}=6.0,\\ \text{Both slabs:} & \;\text{Re}\left\{\epsilon_{yy}\right\}=4.95, \end{array}$$

thereby determining the oscillator strengths via

$$F_{\epsilon }=\left(\epsilon_{\text{target}}-\epsilon_{\infty }\right)\cdot \frac{|\omega_{0}^{2}-\omega_{d}^{2}-i\Gamma \omega_{d}{|}^{2}}{\text{Re}\;\left(\omega_{0}^{2}-\omega_{d}^{2}-i\Gamma \omega_{d}\right)},$$

where *ω*_*d*_ = 2*πc*_0_/*λ*_0_. The chirality strength *F*_*α*_ is computed in the same way from the target value *α*(*ω*_*d*_) = *α*_*c*_ = 2.228 that satisfies the spectral-tuning condition.

This dispersion model ensures that the target values for the medium parameters are met exactly at *λ*_0_, while remaining physically causal and passive away from resonance.

## Appendix D: Material loss and noise

In all simulations the chirality parameter *α* is taken purely real, while losses are introduced through small imaginary parts of the refractive indices ${\bar{n}}_{j}={\bar{n}}_{j}^{\prime }+in_{j}^{\prime \prime }$ with $n_{j}^{\prime \prime }\ll {\bar{n}}_{j}^{\prime }$ . Including absorption renders the refractive indices complex, thereby invalidating the exact tuning condition. This induces both amplitude and phase imbalance between the counter-propagating modes, whence lifting the reflection zero. Nevertheless, for realistic losses the stop-band regions are only slightly modified (see inset of Figure 1 in Ref. [25]), the resonance condition becomes $Re\left(\alpha \right)≃Re\left(\bar{n}\right)$ , and the reflection minimum retains essentially the same spectral position and quadratic *k*_⊥_ dependence. A first-order expansion about the lossless condition gives $|r_{\text{RR}}{|}^{2}\approx c_{\phi }^{2}\Delta \phi^{2}+c_{\ell }^{2}{\left(k_{0}L\;\Delta n^{\prime \prime }\right)}^{2}$ , where Δ*ϕ* = *k*_0_Δ*n*_21_Δ*L* and $\Delta n^{\prime \prime }=n_{2}^{\prime \prime }-n_{1}^{\prime \prime }$ ; *c*_*ϕ*_ and *c*_*ℓ*_ are positive constants. Equal losses in both slabs ( $n_{1}^{\prime \prime }=n_{2}^{\prime \prime }$ ) preserve the reflection zero to first order but reduce the overall contrast. As further evidenced by Figure 6, for loss tangents up to 10^−2^, the minimum reflectance remains significantly low and the parabolic-fit metric degrades only marginally, confirming that moderate absorption reduces contrast without impairing functionality. Even for loss tangents as high as 10^−1^, the shift of the reflection minimum remains below 3 %, and when the two chiral layers exhibit comparable losses, the minimum reflectance can still reach very low values, demonstrating the inherent robustness of the destructive-interference condition. Panel (c) of Figure 6 presents the corresponding parabolic fitting parameter *M*, computed at each tracked reflection dip over *k*_*x*_/*k*_0_ ∈ [−0.6, 0.6] as in Figure 5 in the main text. The fit remains well preserved for comparable losses up to about 5 %, confirming that the Laplacian curvature of |*r*_RR_| is largely preserved. Finally, we note that as with any metamaterial design, losses represent an important limiting factor – particularly at optical frequencies – and must be carefully managed to preserve performance [58].

To assess noise robustness, we propagated additive white Gaussian noise through the reflection transfer function *R*(**k**_⊥_). By comparing the noiseless and noisy outputs, we evaluated the image-domain signal-to-noise ratio (SNR) and its distribution across low- and high-spatial-frequency bands. As shown in Figure 7, the differentiator strongly suppresses low-*k* components associated with the image pedestal while maintaining the high-*k* SNR that governs edge contrast. This behaviour demonstrates that the spectral-hole mechanism redistributes dynamic range toward high spatial frequencies, thereby enhancing edge visibility without appreciable noise amplification.
